# The Influence of Anticipation of Movement Starting Time on Feedforward Activation of Trunk Muscles during Rapid Shoulder Joint Movements

**DOI:** 10.2478/hukin-2022-000073

**Published:** 2022-11-08

**Authors:** Tomoki Oshikawa, Gen Adachi, Hiroshi Akuzawa, Yu Okubo, Koji Kaneoka

**Affiliations:** 1 Faculty of Sport Sciences, Waseda University, Tokyo, Japan.; 2 Waseda Institute for Sport Sciences, Waseda University, Saitama, Japan.; 3 Institute for Human Movement and Medical Sciences, Niigata University of Health and Welfare, Niigata, Japan; 4 Faculty of Health & Medical Care, Saitama Medical University, Saitama, Japan.

**Keywords:** core muscle, onset of electromyographic activity, lumbar spine, lumbar stability

## Abstract

This study aimed to clarify the differences in the onset of trunk muscle activity with and without anticipation of the movement starting time during rapid shoulder movements. Ten healthy men in a relaxed upright position performed rapid 135° flexion, 135° abduction, and 45° extension of the shoulder on the dominant hand side with and without anticipation of the movement starting time. They moved their shoulder joints following a 3-s countdown and a light stimulus in the anticipation and non-anticipation conditions, respectively. Electromyography of the anterior and posterior quadratus lumborum, transversus abdominis, internal oblique, external oblique, rectus abdominis, lumbar multifidus, lumbar erector spinae on the non-dominant hand side, and the middle deltoid on the dominant hand side were measured. The onset of activity of each trunk muscle relative to the onset of the middle deltoid was calculated. Two-way analysis of variance (eight trunk muscles × two anticipation conditions) was used to compare the onset of electromyographic activity of the trunk muscles in each direction of the shoulder movement. There were significant interactions between the muscles and anticipation conditions during shoulder abduction and extension. The onset of activity in the anterior and posterior quadratus lumborum, transversus abdominis, and internal oblique occurred earlier with anticipation of the movement starting time than without anticipation during shoulder abduction and extension. The anticipation of movement starting time may contribute to a reliable center of mass control within the support base and improve lumbar spine stability by hastening the onset of activity of the deep trunk muscles.

## Introduction

Increased activity of each trunk muscle increases intra-abdominal pressure and contributes to lumbar spine stability (Hodges et al., 2005; Vera-Garcia et al., 2007; Imai et al., 2010; Heinen et al., 2015). In addition to the electromyographic (EMG) amplitude of trunk muscle activity, the onset of EMG activity is reportedly useful in investigating trunk muscle activity for lumbar spine stability (Allison et al., 2008; Okubo et al., 2013). The most notable reports on the onset of EMG activity in investigating trunk muscles state that the onset of activity of the transversus abdominis (TrA) is earlier than that of the internal oblique (IO), external oblique (EO), rectus abdominis (RA), and lumbar multifidus (LMF) during rapid upper and lower limb movements (Hodges and Richardson, 1997a, 1997b). Some trunk muscles show a feedforward activation immediately before or after the onset of EMG activity of limb muscles in order to control the center of mass within the support base and stabilize the lumbar spine during rapid limb movements. Several researchers have investigated the onset of TrA activity during rapid limb movements (Allison et al., 2008; Hall et al., 2009; Tsao and Hodges, 2007). TrA has been studied extensively because it attaches to the transverse processes of the lumbar spine through the thoracolumbar fascia (Barker et al., 2006; Macintosh et al., 1987; Tesh et al., 1987).

Quadratus lumborum (QL) directly attaches to the transverse processes of the lumbar spine and has three discrete layers (anterior [QL-a], middle, and posterior [QL-p] layers; Phillips et al., 2008). Each layer is involved in the mechanical control of the pelvis and lumbar spine. Park et al. (2014) investigated the onset of activity of the QL-a and QL-p during rapid shoulder flexion and extension. They revealed that the QL-a acted as a trunk flexor or ipsilateral rotator depending on the direction of the shoulder joint movement. On the other hand, the onset of EMG activity of the QL-p showed no difference depending on the direction of the shoulder joint movement (Park et al., 2014). Oshikawa et al. (2020) reported that the onset of activity of the QL-a and QL-p was earlier than that of the IO, EO, RA, LMF, and lumbar erector spinae (LES) during rapid shoulder abduction and extension. Similar to the TrA, the QL-a and QL-p have also been investigated as muscles in which the onset of EMG activity precedes that of the other trunk muscles.

In previous studies investigating the onset of trunk muscle activity, rapid shoulder movements were adopted as experimental tasks in a situation in which participants did not anticipate a movement starting time in response to visual stimuli (Oshikawa et al., 2020; Park et al., 2014) or auditory stimuli (Allison et al., 2008; Hall et al., 2009; Tsao and Hodges, 2007). An anticipation of perturbation starting time influences the onset of trunk muscle activity (Milosevic et al., 2016; Wilder et al., 1996). Milosevic et al. (2016) reported that the onset of activity of the RA and LES was earlier with anticipation than without anticipation of perturbation starting time in an external perturbation task in which the support surface moved while participants were sitting. Sudden movement or perturbation without anticipation does not allow sufficient anticipatory postural adjustment prior to movement and can result in low back pain. However, it has not been clarified whether the onset of trunk muscle activity, including the TrA, IO, and QL, is altered with and without anticipation of movement starting time during internal perturbation tasks. Therefore, it is important to compare the onset of trunk muscle activity during rapid shoulder movements as an internal perturbation task with and without anticipation of movement starting time in order to verify the trunk muscles that are related to anticipatory postural adjustment. If it becomes clear which trunk muscles are influenced by anticipation, it will lead to the advancement of approaches to improve lumbar spine stability and postural control. For example, in order to improve trunk muscles at the start of limb movement for walking and lifting in daily life, and at the start of track and field sprinting in sports, it is a priority to approach the trunk muscles in which the activity onset is accelerated by anticipation.

This study aimed to clarify the differences in the onset of trunk muscle activity with and without anticipation of movement starting time during rapid shoulder movements. We analyzed the onset of activity of eight trunk muscles and the middle deltoid (MD), which is the prime mover of the shoulder during rapid shoulder flexion, abduction, and extension.

## Methods

### Participants

Ten healthy men (mean ± standard deviation [SD] age, 23 ± 1 years; body height, 174.8 ± 4.6 cm; body mass, 73.0 ± 8.3 kg) participated in this study. We excluded participants who had experienced low back pain or lower limb injury within the last 5 years or who had a history of spinal or lower limb surgery. None of the participants had any specific sports or training habits and no participant was engaged in work involving heavy loads or perturbation on the trunk. Written informed consent was obtained from all participants. The experimental protocol followed the Declaration of Helsinki and was approved by the ethics committee of our institution.

### Measures

#### Electromyography

**Figure 1:**
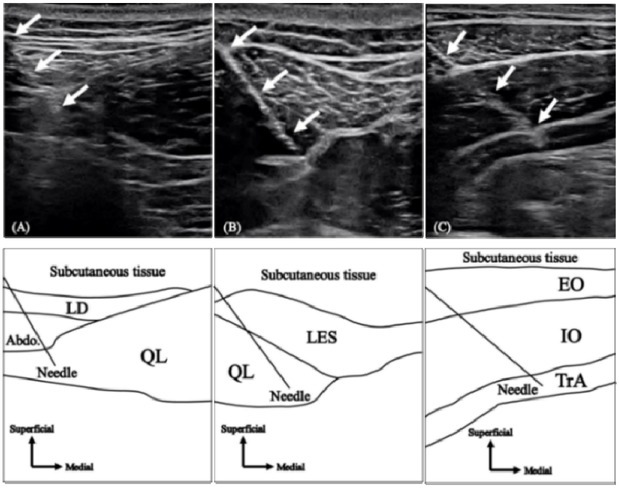
Hypodermic needle insertion Ultrasound images (upper) and line drawings (lower) of the hypodermic needle insertion to (A) anterior and (B) posterior layers of the quadratus lumborum and (C) transversus abdominis. White arrows in the ultrasound images indicate the full needle shaft. Line drawings show a guide to ultrasound images. QL, quadratus lumborum; TrA, transversus abdominus; LD, latissimus dorsi; Abdo, abdominal muscles; LES, lumbar erector spinae; EO, external oblique; IO, internal oblique. (Oshikawa et al., 2020a).

EMGs of the QL-a, QL-p, and TrA were recorded using intramuscular fine-wire electrodes, while those of the IO, EO, RA, LMF, LES, and MD were recorded using surface electrodes. Trunk muscle activities were measured on the non-dominant hand side of participants, and the MD activity was measured on the dominant hand side. Bipolar intramuscular fine-wire electrodes (Unique Medical Co., Ltd., Tokyo, Japan) were formed from two 0.08-mm strands of urethane-coated stainless steel wires, wherein urethane was removed from the end. The fine-wire was threaded into a 23-gauge hypodermic needle (QL: 0.65 × 90 mm, TrA: 0.60 × 60 mm) with tips bent to form 3- and 5-mm hooks. The fine wire and needle were sterilized at 121°C for 20 min in an autoclave. Fine-wire electrodes were inserted into the target muscles using ultrasound imaging (SONIMAGE HS1 PRO, KONICA MINOLTA Co., Ltd., Tokyo, Japan) by an experienced orthopedic doctor. The QL-a electrodes were inserted approximately 5 cm lateral to the spinous process at the third or the fourth lumbar (L3/4) level and placed near the lateral border of the QL (Figure 1A). The QL-p electrodes were inserted 4 cm lateral to the spinous process at the L3/4 level and placed near the medial border of the QL (Figure 1B) (Oshikawa et al., 2020a, 2020b; Park et al., 2012, 2013). The TrA electrodes were inserted 2 cm medial to the midpoint of the line from the anterior superior iliac spine to the rib cage (Figure 1C) (Hodges and Richardson, 1997b). Each insertion point was adjusted using ultrasound imaging, where each muscle was clearly described for each participant. For more than 2 years after the experiment, there were no adverse events due to the insertion of the intramuscular fine-wire electrodes.

Before the attachment of the surface electrodes, the skin was abraded with a skin abrasive and alcohol was applied to reduce skin impedance to < 2 kΩ. Surface electrodes (BlueSensor N-00-S, METS Co., Tokyo, Japan) with an 8-mm diameter were attached to each muscle belly, which were parallel to the muscle fiber; for the IO, 1 cm medial and downward to the anterior superior iliac spine (Ng et al., 1998); EO, 15 cm lateral to the umbilicus (Okubo et al., 2010b); RA, 3 cm lateral to the umbilicus (Okubo et al., 2010b); LMF, 2 cm lateral to the L5 spinous process (Okubo et al., 2010a); LES, 3 cm lateral to the L3 spinous process (Okubo et al., 2010b); and MD, the midpoint between the acromion and deltoid tuberosity (Oshikawa et al., 2020). The inter-electrode distance was 20 mm. A wireless EMG telemeter system (BioLog DL-5000, S&ME Co., Tokyo, Japan) with a sampling rate of 2000 Hz was used to measure both fine-wire and surface EMGs.

### Design and Procedures

#### Design

The present study used a repeated measurement design to compare the onset of EMG activity with and without anticipation of the movement starting time and among the trunk muscles. The independent variables were rapid shoulder joint flexion, abduction, and extension movements with and without anticipation of the movement starting time. The dependent variable was the onset of EMG activity of the trunk muscles relative to the onset of MD activity during rapid shoulder joint movements. This study was conducted in a laboratory from April to October 2018.

#### Procedures

In relaxed upright positions, participants performed rapid 135° flexion, 135° abduction, and 45° extension of the shoulder on the dominant hand side with and without anticipation of the movement starting time. In the shoulder joint movements with anticipation of the movement starting time, participants moved their shoulder joint following a 3-s countdown by the examiner. The countdown was performed according to the metronome rhythm set at 60 beats/min. The timing of the count “1” by the examiner was recorded in the EMG data using the trigger signal. In the shoulder joint movements without anticipation of the movement starting time, participants moved their shoulder joint after a light stimulus. The light was placed 3 m ahead of participants, and the timing of the light emission was recorded in the EMG data by the trigger signal. Participants practiced three to five times before each trial was recorded. Each trial was recorded three times, while the examiners confirmed the relaxed condition of participants on the EMG monitor during the countdown and before the light stimulus. The trials were randomly conducted. The rest interval between each trial was at least 1 min.

### Data analysis

The onset of EMG activity was calculated based on the methods described in a previous study (Oshikawa et al., 2020). The recorded EMG data were analyzed using biomedical information software (BIMUTAS-Video, Kissei Comtec Co., Ltd., Nagano, Japan). Raw EMG data were bandpass filtered between 10 and 950 Hz. After full-wave rectification of filtered data, the mean and SD of the EMG amplitude in the relaxed standing for 50 ms before each count “1” or a light stimulus were calculated. Then, the threshold of the onset of EMG activity was set at “mean + 2 SD for 50 ms in relaxed standing”. Linear envelopes of each muscle were created from the rectified EMG data using a 40-data-point (20 ms integrated EMG) moving average (Allison et al., 2008; Oshikawa et al., 2020). The first point where the mean EMG amplitude exceeded the threshold continuously for at least 20 ms was defined as the onset of EMG activity. The examiners confirmed the EMG waveform for the defined onset, both visually and numerically. For instance, when the amplitude exceeded the threshold before or during the countdown and before the light stimulus, the highest single amplitude was not defined as the onset of EMG activity (Allison et al., 2008; Okubo et al., 2013; Oshikawa et al., 2020). The onset of the activity of each trunk muscle relative to the onset of the MD activity was calculated from the onset of each muscle activity. The mean onset time of the three trials was used for statistical analyses as a representative value for each participant. A previous study (Hodges and Richardson, 1997a) showed that there were no differences in the onset of EMG activity between the fine-wire and surface electrodes.

### Statistical Analysis

SPSS Statistics 25.0 (IBM Japan Co., Ltd., Tokyo, Japan) was used for statistical analyses. The reliability of the onset of EMG activity of the trunk muscles relative to that of the MD was assessed using the intraclass correlation coefficient (ICC), with values of <0.4, 0.4-0.59, 0.60-0.74, and ≥0.75, indicating poor, fair, good, and excellent, respectively (Fleiss et al., 2013). After confirming all data for normal distribution by Kolmogorov-Smirnov tests and for homoscedasticity by Levene’s tests, two-way analysis of variance (ANOVA) (8 trunk muscles × 2 anticipation conditions) was used to compare the onset of EMG activity of the trunk muscles relative to that of the MD in each direction of shoulder joint movement. Regarding the onset of the RA during shoulder flexion and abduction and that of the LMF and LES during shoulder extension, the onset of EMG activity was observed in six or fewer participants. Therefore, the data from the RA during shoulder flexion and abduction and the LMF and LES during shoulder extension were excluded from all statistical analyses. The alpha level was set at 0.05. A Bonferroni correction was applied for post-hoc tests to examine the main effects and simple main effects on the trunk muscle factors. Since SPSS Statistics 25.0 calculates the "adjusted *p*-value" by multiplying the original *p*-value by 21 (= _7_C_2_) for shoulder flexion and abduction and by 15 (= _6_C_2_) for shoulder extension, the adjusted *p*-values are shown in the results section. Partial *η^2^* was calculated to estimate the effect size for the two-way ANOVA, with values of 0.01-0.06, 0.06-0.14, and ≥0.14, indicating small, medium, and large effects, respectively (Cohen, 1988).

## Results

**Figure 2:**
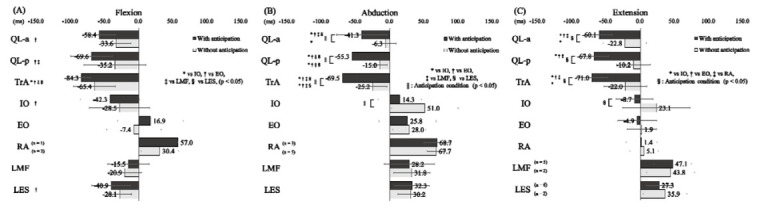
Onset of trunk muscle activity Onset of trunk muscle activity relative to that of the middle deltoid during rapid shoulder flexion (A), abduction (B), and extension (C). Data are expressed as mean ± standard deviation. Zero indicates the onset of the middle deltoid. QL-a, quadratus lumborum anterior layer; QL-p, quadratus lumborum posterior layer; TrA, transversus abdominis; IO, internal oblique; EO, external oblique; RA, rectus abdominis; LMF, lumbar multifidus; LES, lumbar erector spinae.

The onset of EMG activity (mean ± SD) of each trunk muscle relative to the onset of the MD in each trial is displayed in Figure 2. Table 1 shows a breakdown of the number of participants in the top three muscles that showed the fastest onset of activity in each trial. The ICCs of the onset of EMG activity of the trunk muscles relative to that of the MD during shoulder flexion, abduction, and extension ranged from 0.791 to 0.975, 0.778 to 0.941, and 0.805 to 0.929, respectively. In shoulder flexion (Figure 2A), there was no significant interaction between the muscles and anticipation conditions (F_6, 126_ = 1.739, *p* = 0.117, partial *η^2^* = 0.076), and no significant main effect of anticipation conditions (F_1, 126_ = 3.612, *p* = 0.060, partial *η^2^* = 0.028). However, there was a significant main effect of the muscles (F_6, 126_ = 11.542, *p* < 0.001, partial *η^2^* = 0.355). The onsets of the QL-a (with anticipation: -58.4 ± 23.9 ms, without anticipation: -33.6 ± 22.2 ms), IO (with anticipation: -42.3 ± 43.5 ms, without anticipation: -28.5 ± 44.1 ms), and LES (with anticipation: -40.9 ± 28.3 ms, without anticipation: -28.1 ± 17.3 ms) were significantly earlier than that of the EO (with anticipation: 16.9 ± 48.2 ms, without anticipation: - 7.4 ± 39.7 ms) (*P* < 0.007). The onset of the QL-p (with anticipation: -69.6 ± 30.6 ms, without anticipation: -35.2 ± 45.6 ms) was significantly earlier than that of the EO and LMF (with anticipation: -15.5 ± 30.4 ms, without anticipation: -20.9 ± 24.8 ms) (*P* < 0.033). Furthermore, the onset of the TrA (with anticipation: -84.3 ± 13.0 ms, without anticipation: -65.4 ± 30.7 ms) was significantly earlier than that of the IO, EO, LMF, and LES (*p* < 0.006).

**Table 1:** A breakdown of the number of participants in the top three muscles that showed the fastest onset of activity in each trial.

Trial	Onset order	Quadratus lumborum anterior layer	Quadratus lumborum posterior layer	Transversus abdominis	Internal oblique	External oblique	Rectus abdominis	Lumbar multifidus	Lumbar erector spinae
Flexion	1		4	6					
with anticipation	2	2	2	3	3				
	3	5		1	2				2

Flexion	1		1	7	1				1
without anticipation	2	2	5	2					1
	3	4	2	1	2				1

Abduction	1		3	7					
with anticipation	2	5	3	2					
	3	2	4	1	2	1			

Abduction	1		2	7	1				
without anticipation	2	2	6	2					
	3	8	2						

Extension	1	1	5	4					
with anticipation	2	3	2	4		1			
	3	5	3	2					

Extension	1	5	2	3					
without anticipation	2	2	3	3		2			
	3	1	2	3	1	2	1		

e.g.: In flexion with anticipation, 6 out of 10 participants showed the fastest onset of the transversus abdominis, and 4 out of 10 participants showed the fastest onset of the quadratus lumborum posterior layer. Also, 3 out of 10 participants showed the second fastest onset of the transversus abdominis and internal oblique, and 2 out of 10 participants showed the second fastest onset of the quadratus lumborum anterior and posterior layer. Additionally, 5 out of 10 participants showed the third fastest onset of the quadratus lumborum anterior layer, and 2 out of 10 participants showed the third fastest onset of the internal oblique and lumbar erector spinae, and 1 out of 10 participants showed the third fastest onset of the transversus abdominis.

In shoulder abduction (Figure 2B), there was a significant interaction between the muscles and anticipation conditions (F_6, 126_ = 2.483, *P* = 0.026, partial *η^2^* = 0.106). The onset of the QL-a (41.3 ± 36.8 ms), QL-p (-55.3 ± 22.3 ms), TrA (-69.5 ± 21.2 ms), and IO (14.3 ± 31.7 ms) with anticipation of the movement starting time was significantly earlier than that of the QL-a (-6.3 ± 15.4 ms), QL-p (-15.0 ± 10.8 ms), TrA (-25.2 ± 20.8 ms), and IO (51.0 ± 48.6 ms) without anticipation (*p* < 0.009). For the condition of added anticipation of the movement starting time, the onset of the QL-a, QL-p, and TrA was significantly earlier than that of the IO, EO (25.8 ± 41.9 ms), LMF (28.2 ± 36.7 ms), and LES (32.3 ± 25.1 ms) (*p* < 0.001). When no anticipation was added, the onset of QL-a was significantly earlier than that of IO (*p* < 0.001). In addition, the onset of the QL-p and TrA was significantly earlier than that of the IO, EO (28.0 ± 24.3 ms), LMF (31.8 ± 26.5 ms), and LES (30.2 ± 18.6 ms) (*p* < 0.026).

In shoulder extension (Figure 2C), there was a significant interaction between the muscles and anticipation conditions (F_5, 108_ = 3.089, *p* = 0.012, partial *η^2^* = 0.125). The onset of the QL-a (60.1 ± 18.9 ms), QL-p (-67.8 ± 21.1 ms), TrA (-71.0 ± 23.2 ms), and IO (-8.7 ± 27.0 ms) with anticipation of the movement starting time was significantly earlier than that of the QL-a (-22.8 ± 31.5 ms), QL-p (-10.2 ± 24.6 ms), TrA (-22.0 ± 31.3 ms), and IO (23.1 ± 49.2 ms) without anticipation (*p* < 0.012). For the condition of added anticipation, the onset of the QL-a, QL-p, and TrA was significantly earlier than that of the IO, EO (-4.9 ± 28.7 ms), and RA (1.4 ± 22.7 ms) (*p* < 0.001). When no anticipation was added, the onset of the QL-a and TrA was significantly earlier than that of the IO (*p* < 0.007).

## Discussion

This study compared the onset of trunk muscle activity during rapid shoulder movements with and without anticipation of movement starting time. The present study is the first to reveal the influence of anticipating the movement starting time of the internal perturbation task on the onset of trunk muscle activity. The body always requires postural control against internal perturbation caused by movements of the limbs, even in daily living. The findings of the present study showed that the onset of the QL-a, QL-p, TrA, and IO was accelerated by the anticipation of the internal perturbation timing. These muscles play important roles in stabilizing the lumbar spine and controlling the posture during anticipated movements in healthy individuals.

The onset of the QL-a, QL-p, TrA, and IO occurred earlier with anticipation than without anticipation during shoulder abduction and extension. In addition, although not significant, the onset of these muscles was earlier with anticipation than without anticipation during shoulder flexion. Regarding rapid shoulder movements, Park et al. (2014) proposed a model for trunk movements resulting from reactive moments produced by rapid shoulder movements. According to this model, rapid shoulder movements generate reactive moments of trunk lateral flexion to the shoulder movement side. The QL-a and QL-p function in ipsilateral trunk flexion (Park et al., 2012, 2013). The results of the present study showed that the onset of the QL-a and QL-p occurred earlier in any direction of shoulder movement due to the anticipation of movement starting time, implying that the QL-a and QL-p may decrease the perturbation produced by the reactive moments of trunk lateral flexion to the shoulder movement side. The TrA and IO are the muscles involved in increasing intra-abdominal pressure (Cresswell and Thorstensson, 1994; Tayashiki et al., 2016). We speculate that the onset of the TrA and IO occurred earlier in order to “pre-increase” the intra-abdominal pressure for lumbar spine stability when moving the shoulder rapidly in response to the examiner’s countdown. The results of the QL-a, QL-p, TrA, and IO indicate that the anticipation of movement starting time contributes to the reliable center of mass control within the support base and improves lumbar spine stability by accelerating the onset of activity of the muscles located deep in the trunk. In addition, we clarified that the early onset of the QL-a, QL-p, TrA, and IO was an ideal response to internal perturbation due to rapid shoulder movements. Since the onset of the TrA in patients with low back pain during rapid shoulder movements without anticipation of movement starting time was slower than that in healthy participants (Hodges and Richardson, 1996), it is important to activate the TrA in advance of sudden movements in order to prevent and improve low back pain. Additionally, TrA isolated training and TrA, IO, EO, and RA co-activation training for low back pain patients partially hastened the onset of the abdominal muscles during rapid shoulder movements without the anticipation of movement starting time (Tsao and Hodges, 2007). Therefore, exercises aimed at activating the QL-a, QL-p, TrA, and IO may be important for ideal postural adjustment and prevention of or decreasing low back pain. For example, the implementation of the side bridge, which activates the QL-a, QL-p, TrA, and IO (Oshikawa et al., 2021), may hasten the onset of these muscles during rapid shoulder movements without the anticipation of movement starting time. Presumably, this will lead to reliable postural adjustment and improvement in lumbar spine stability during sudden movements.

Regarding the onset between the trunk muscles, the onset of the TrA was earlier than that of the IO, EO, LMF, and LES during shoulder flexion, regardless of the anticipation. During shoulder abduction, the onset of the QL-a, QL-p, and TrA was earlier than that of the IO, EO, LMF, and LES with anticipation, and the onset of the QL-p and TrA was earlier than that of the IO, EO, LMF, and LES without anticipation. Additionally, the onset of the QL-a, QL-p, and TrA was earlier than that of the IO, EO, and RA during shoulder extension with anticipation. Oshikawa et al. (2020) reported that the onset of the QL-a, QL-p, and TrA was earlier than that of the IO, EO, RA, LMF, and LES during rapid shoulder movements, which were similar to the tasks without anticipation in the present study. The early onset of the QL-p and TrA can improve lumbar spine stability because the QL-p attaches directly to the transverse processes of the lumbar spine at the L1-4 level (Phillips et al., 2008) and the TrA attaches to the transverse processes of the lumbar spine through the thoracolumbar fascia (Barker et al., 2006). Considering the model for trunk movements resulting from the reactive moments produced by rapid shoulder movements (Park et al., 2014), we speculate that the early onset of the QL-a, an ipsilateral trunk flexor, was also involved in the center of mass control within the support base.

Although high SDs were observed in the onset of almost all muscles, Table 1 shows that the onset of the QL-a, QL-p, and TrA was early in many participants regardless of the anticipation. This suggests that the onset order is approximately the same for each participant, and the high standard deviations may be due to differences in the extent between the participants. Hodges and Richardson (1997b) reported that differences in the relative placement of electrodes for each participant might cause large variability among participants. In addition, differences in the standing posture of each participant may have been related to the large variation among participants. In the results of the present study, SD was in the range of ± 11-49 ms and standard error (SE) (= SD / √10 [participant number]) was in the range of ± 3-16 ms. These values are equal to or smaller than those of previous studies evaluating the onset of trunk muscles during rapid shoulder movements (SD: ± 18-104 ms [Hodges and Richardson, 1996], SE: ± 6-23 ms [Hodges and Richardson, 1997b]).

The onsets of the RA during shoulder flexion and abduction and of the LMF and LES during shoulder extension were not observed in some participants. Hodges and Richardson (1997b) and Oshikawa et al. (2020) reported that trunk muscles such as the RA, LMF, and LES had direction-specific responses during rapid shoulder movements. Therefore, we speculate that the feedforward activation of the RA, LMF, and LES was not required for specific movement directions in each muscle. During shoulder flexion, the onsets of the EO, RA, and LMF were later during the condition with anticipation compared to the condition without anticipation. Regarding the EO, since few participants showed the RA onset, we considered that the superficial abdominal muscles such as the EO and RA showed only a small contribution to postural adjustment during shoulder flexion. Therefore, we suggest that the EO did not have a significant influence on postural adjustment, even if the EO onset was delayed during the condition with anticipation. With regard to the RA, one participant who showed the RA onset during the condition with anticipation and two participants who showed the RA onset during the condition without anticipation could not be compared because they were different participants. We consider that the difference of 5.4 ms in the LMF onset with and without anticipation is clinically non-significant.

There are some limitations of the present study. First, participants were only healthy men. If participants were women and/or subjects with low back pain, the results might have been different. Second, we did not measure the activity of the anterior and posterior deltoid muscles as the upper limb muscles. EMG activity of the anterior and posterior deltoid muscles should have been measured as the prime movers of shoulder flexion and extension, respectively. The present study could not compare the onset of trunk muscle activity during shoulder movement in different directions. This study considered shoulder movement, which has been used in many previous studies. However, most movements in daily life and sports begin in the lower, not the upper limbs. Therefore, future studies should examine the influence of anticipating the movement starting time on the onset of trunk muscle activity during lower-limb movements. Additionally, it would be interesting to examine the influence of anticipating the movement starting time on the onset of trunk muscle activity in complex activities, such as walking and sports, rather than simple shoulder movement tasks.

In conclusion, the present study investigated the onset of trunk muscle activity during rapid shoulder movements with and without anticipation of movement starting time. The anticipation of movement starting time hastened the onset of the QL-a, QL-p, TrA, and IO during rapid shoulder abduction and extension. Rehabilitation exercises for activating the QL-a, QL-p, TrA, and IO may lead to reliable postural adjustment and improvement of lumbar spine stability during sudden movements.
